# Missed diagnosis of psychosis in a patient with Autism Spectrum Disorder: A Case Report

**DOI:** 10.1192/j.eurpsy.2026.12121

**Published:** 2026-07-31

**Authors:** Y. T. Yap

**Affiliations:** General Psychiatry, Institute of Mental Health, Singapore, Singapore

## Abstract

**Introduction:**

Autism spectrum disorder (ASD) is a neurodevelopmental disorder. The diagnosis of comorbid psychotic disorder requires detailed clinical assessment and its early identification and interventions are important in improving clinical outcomes.

**Objectives:**

We present a case of ASD who was initially diagnosed with behavioural issues related to ASD, but subsequently diagnosis revised to psychosis. We aim to highlight the diagnostic challenges of psychosis in ASD.

**Methods:**

Details of the case were described. Information was gathered based on medical records.

**Results:**

Mr S, a 24-year old Caucasian, was first brought to our hospital’s emergency services by the police for a possible case of unsound mind. Patient was travelling in Singapore, and he was noted to be behaving abnormally and shouting and disturbing others in public, and his mother was unable to manage his behaviour and hence patient was brought in by the police for assessment. Patient has a past medical history of Autism Spectrum Disorder for which he is on oral aripiprazole 2.5mg OM, and has only been partially compliant since coming to Singapore. Patient was admitted to the ward with the impression that patient’s behaviour is related to his autism spectrum disorder. Mother corroborated that patient has been behaving differently from his usual self in the past 1 week. Patient was noted to be paranoid towards phone scammers and police, and have been exhibiting more disruptive and potentially dangerous behaviour as well. During his admission, patient’s behaviour and mental state was closely monitored and he was noted to have grandiose and bizarre delusions that that he is a “Wing-Chun Master” and is the main lead actor and producer of a movie film in the ward. He also declined to be addressed by his name for fear of being captured by CCTVs in the ward. Diagnosis was then revised to brief psychotic disorder and he was treated accordingly.

**Conclusions:**

Diagnosing psychosis in individuals with Autism Spectrum Disorder (ASD) presents considerable challenges due to overlapping symptomatology. Even experienced psychiatrists may encounter difficulty distinguishing between the two conditions in certain cases. In addition to obtaining a detailed corroborative history from family members, it is essential to conduct close and ongoing monitoring of the patient’s symptoms and mental state before establishing a definitive diagnosis. This is particularly important as management strategies may differ significantly depending on the diagnosis, and timely intervention is crucial for optimising clinical outcomes.

**Disclosure of Interest:**

None Declared

